# Chemical and Biological Changes Under Force Degradation and Acceleration Condition of the Combination of Ha-Rak Remedy, *Piper betle,* and *Garcinia mangostana* Extracts for Atopic Dermatitis

**DOI:** 10.1155/2024/4297596

**Published:** 2024-10-28

**Authors:** Ubonwan Saesiw, Srisopa Ruangnoo, Arunporn Itharat, Pattama Sriumpai

**Affiliations:** ^1^Philosophy Program in Department of Applied Thai Traditional Medicine, Faculty of Medicine, Thammasat University, Klongluang, Pathumthani 12120, Thailand; ^2^Department of Applied Thai Traditional Medicine, Faculty of Medicine, Thammasat University, Klongluang, Pathumthani 12120, Thailand; ^3^Center of Excellence for Applied Thai Traditional Medicine Research (CEATMR) in Thammasat University Research Division, Thammasat University, Klongluang, Pathumthani 12120, Thailand

**Keywords:** accelerated study, antiallergy, anti-inflammation, forced degradations, HPLC, stability study

## Abstract

Herbal medicine could be an option for atopic dermatitis (AD) treatment for those suffering from global public health. HMB is a new combination of three herb extracts, consisting of the Ha-Rak (HR) remedy extract, *Piper betle* (PB) extract, and *Garcinia mangostana* (GM) extract in equal proportions, using Thai traditional medicine theory, that uses a combination of medications that can improve therapeutic efficacy and reduce side effects and toxicity. HMB extract has anti-inflammatory and antiallergic properties, is a component for AD treatment, and tends to develop topical products. Drug registration requires stability data. Results from drug stability testing affect not only the efficacy of the drug but also its safety. The aim of this study was to investigate stability through forced degradation and an accelerated study of extracts. Chemical content analysis and *in vitro* biological activities such as anti-inflammatory and antiallergic activities determined the effects of all examined samples. Anti-inflammatory and antiallergic effects were assessed by inhibiting nitric oxide synthesis in RAW 264.7 cells and *β*-hexosaminidase release in RBL-2H3 cells, respectively. High-performance liquid chromatography (HPLC) assessed content indicators. Moisture and temperature hydrolysis had no significant differences in the chemical or biological properties of the HMB. However, the HMB demonstrated sensitivity to alkaline hydrolysis, showed low anti-inflammatory activity, and decreased hydroxychavicol, eugenol, and *α*-mangostin contents. The contents of the three compounds also decrease with acid hydrolysis. For the accelerated study, anti-inflammatory and antiallergic effects and hydroxychavicol amount were not significantly different after 180 days at 40°C and 75% RH. Therefore, the contents of eugenol and *α*-mangostin were changed. Eugenol in HMB decreased significantly from the 15^th^ day until the 180^th^ day of storage. In addition, *α*-mangostin amounts in HMB decreased slightly on 180^th^ day. Fortunately, reducing the two chemicals did not affect anti-inflammatory or antiallergic effects. For stability, combination extract should be stored in a closed container in the refrigerator at a low temperature and protected from light, high temperature, oxygen, and pH. Further HMB development should avoid pH or oxidation processes or components.

## 1. Introduction

Atopic dermatitis (AD) is a widespread chronic relapsing skin condition that is highly prevalent, ranks 15^th^ among all nonfatal diseases of the population worldwide, and is a major public health problem worldwide [1–3]. AD can have a significant impact on the daily lives of patients, carers, and families, as well as significant financial consequences for individuals and societies [4]. AD is a type I immunological reaction mediated by immunoglobulin (Ig) E, generated by B cells and other plasma cells. These antibodies attach to mast cell or basophil surface membranes, causing them to degranulate and produce inflammatory mediators and allergy-related cytokines, including nitric oxide, histamine, and *β*-hexosaminidase [5–8]. Meanwhile, topical corticosteroids, a first-line medication, can have major side effects such as skin thinning, stretch marks, glaucoma and cataracts, adrenal suppression, and topical steroid addiction and withdrawal [9].

Complementary and alternative medicines (CAMs) are utilized by more than 80% of the world's population and are becoming an increasingly important component of the healthcare system. Herbal medicine is one of the most widely used CAMs [10]. For centuries, humans have used herbal medicine to treat a variety of illnesses. Three well-known herbal medicines are commonly used as topical drugs in Thai traditional medicine for treating skin disorders such as AD. Ha-Rak remedy (HR), which consists of equal ratios of five plant roots, is used to relieve burning skin [11, 12]. The leaves of *Piper betle* Linn. (PB) are used to relieve itchy skin and skin inflammation. The pericarps of *Garcinia mangostana* Linn. (GM) are used to treat acute and chronic wounds [12]. These plant parts were mentioned in the National List of Essential Medicines [12] and possess specific properties that are associated with AD disease. The previous reports revealed that the 95% ethanolic extract of the HR remedy had an anti-inflammatory effect with an IC_50_ value of 40.36 *μ*g/mL. Pectolinarigenin was isolated from the HR remedy which also had an anti-inflammatory effect. The ethanolic extract of PB also has anti-inflammatory effects by decreasing nitric oxide in murine peritoneal macrophages, as measured by the Griess assay [13]. In addition, the 95% ethanolic extract of betel leaves significantly decreased histamine and granulocyte macrophage colony-stimulating factor (GM-CSF) produced by an IgE-mediated hypersensitivity reaction, as well as inhibited exotoxin and IL-8 secretion in a TNF-*α* and IL-4-induced allergic reaction [14]. Hydroxychavicol, the main component of PB, was found to have anti-inflammatory properties. It has the potential to suppress TNF expression in LPS-stimulated cells. Hydroxychavicol could reduce TNF expression levels compared to control standards. Eugenol inhibited several proinflammatory mediators, including tumor necrosis factor alpha (TNF-*α*) and prostaglandin E_2_ (PGE_2_), inducible expression of nitric oxide synthase (iNOS) [15]. In C6 rat glioma cells, a 95% ethanolic extract of GM pericarps reduced A23187-induced PGE_2_ (an inflammatory cytokine) generation [16]. The *α*-mangostin in GM significantly inhibited lipopolysaccharide-stimulated NO production in RAW 264.7 cells. *α*-Mangostin also significantly reduced PGE_2_ production in lipopolysaccharide-activated RAW 264.7 cells [17].

The HMB, a new combination remedy, consists of HR remedy, PB, and GM extracts. Our previous study (unpublished data) had shown that the combination of HR, PB, and GM extracts (HMB) showed strong anti-inflammatory activity by inhibiting NO production, with an IC_50_ value of 1.73 *μ*g/mL. HMB inhibited *β*-hexosaminidase release from RBL-2H3 cells more than chlorpheniramine, a standard antihistamine, with IC_50_ values of 6.54 and 15.74 *μ*g/mL, respectively. The HMB contained three active compounds: hydroxychavicol, eugenol, and *α*-mangostin. The contents of the three compounds in HMB extract were 121.93, 54.10, and 86.23 mg/g of the extract, respectively, using the high-performance liquid chromatography (HPLC) technique.

The novel remedy that we developed is being turned into pharmaceutical products that will be helpful to AD patients. For drug registration, data on drug stability is necessary. Results from drug stability testing affect not only the efficacy of the drug but also its safety, including the suitability of drug storage, the shelf life of the drug, and the development of further products.

Therefore, the HMB remedy needs to be tested for stability study, including a forced degradation study (stress testing) and an accelerated study. Also, forced degradation and accelerated studies were performed for the development of new drug substances and new drug products. We assessed the in vitro anti-inflammation, antiallergy, and chemical content and reported the results herein. Pectolinarigenin, eugenol, hydroxychavicol, and *α*-mangostin can be markers for the stability control of HMB.

## 2. Materials and Methods

### 2.1. Plant Materials Extraction and Preparation

Each plant of the HR remedy (roots of *Capparis micracantha* DC. [CAPPARIDACEAE], *Clerodendrum petasites* S. Moore [VERBENACEAE], *Ficus racemosa* Linn. [MORACEAE], *Harrisonia perforata* Merr. [SIMAROUBACEAE], and *Tiliacora triandra* Diels. [MENISPERMACEAE]) and the leaves of PB Linn. (PIPERACEAE) and the pericarps of GM Linn. were purchased from Chachoengsao, Pathum Thani Province, Thailand. All plant materials were identified by the Office of the Herbarium of Southern Center of Thai Medicinal Plants at the Faculty of Pharmaceutical Science, Prince of Songkhla University, Songkhla, Thailand, as shown in Table 1.

Each dried plant material was powdered. For the HR remedy extract, five plant powders of the HR remedy were mixed in an equal ratio and macerated three times for 3 days with 95% ethanol at room temperature. Each ethanolic extract of the PB Linn. and GM Linn. was obtained by macerating the powdered plant materials three times for 3 days with 95% ethanol at room temperature. Three macerates were filtered and concentrated to dryness by using a rotary evaporator. Their percentage of yields is shown in Table 1. The three ethanolic extracts were mixed in an equal ratio (the ratio of the HR remedy extract, PB Linn. extract, and GM Linn. extract was 1:1:1).

### 2.2. Forced Degradation Study (Stress Testing)

The forced degradation study was modified from the International Conference on Harmonization (ICH) guidelines [18]. The test consists of five conditions, including temperature, moisture, acid hydrolysis, alkaline hydrolysis, and oxidation. The HMB extract was prepared by weighing up to 50 mg in each test tube, and then the stress agent was added in each condition as follows for the temperature test: the HMB extract was heated at 80°C for 3 h for moisture hydrolysis test, the HMB extract was added to 150 *μ*L of distilled water for acid hydrolysis test, the HMB extract was added to 150 *μ*L of 3 N hydrochloric acid for alkaline hydrolysis test, the HMB extract was added to 150 *μ*L of 3 N sodium hydroxide for oxidation test, and the HMB extract was added to 150 *μ*L of 30% hydrogen peroxide. Then, all tested samples were heated at 80°C for 3 h. The samples were tested for anti-inflammatory activity and studied for drug content analysis by the HPLC technique.

### 2.3. Accelerated Study

The accelerated study was modified from ICH guidelines [19]. The HMB extract was placed during a six-month period, under the acceleration conditions at 40°C ± 2°C with 75% ± 5% RH. The HMB extract was taken at Day 0 (control sample), 15, 30, 60, 90, 120, 150, and 180. After testing, the samples will be determined to have anti-inflammatory activity and studied for drug content analysis by the HPLC technique.

### 2.4. Determination of LPS-Induced NO Production From RAW 264.7 Cells

The inhibitory effects of all tested samples were evaluated by the modified method of Tewtrakul et al. [20]. The RAW 264.7 cells (ATCC TIB-71) were cultured in the RPMI 1640 medium supplemented with 10% heat-inactivated FBS and 1% penicillin and streptomycin (10,000 units/mL of penicillin and 10,000 mg/mL of streptomycin). In 96-well plates, the cells were added at a concentration of 1 × 10^5^ cells/well and incubated for 24 h at 37°C with 5% CO_2_. Then, the medium was removed and 100 *μ*L of LPS (5 ng/mL) was added to each well; subsequently, the cells were treated with various concentrations of samples which were dissolved in DMSO (100 *μ*L/well) and incubated. Griess reagent was used to determine NO production after 24 h. In 96-well plates, 100 *μ*L of the supernatant obtained from each well was combined with an equal volume of Griess reagent (comprising 1% sulfanilamide and 0.1% N-(1-naphthyl) ethylenediamine dihydrochloride in 2.5% phosphoric acid). A microplate reader was used to measure the absorbance at 570 nm.

Thiazolyl blue tetrazolium bromide (MTT) assay was used to determine cell viability in this study. MTT solution at 5 mg/mL concentration (10 *μ*L) was added to each well after transferring the supernatant. The 96-well plates were then incubated for two hours. Following the removal of the media, 100 *μ*L of isopropanol containing 0.04 M of HCl was added to each well to attempt to dissolve the formazan dye within the cells. A microplate reader was used to measure the absorbance at 570 nm. Each well had a 0.2% DMSO concentration. When the sample group's optical density was less than 70% of the control groups, the samples were considered cytotoxic. IC_50_ values were computed from the Prism program, and the inhibition (%) of NO generation was determined by using the following equation:

(1) $$\begin{array}{c} \text{inhibition }\left(\%\right)=\left[\frac{{\text{OD}}_{\text{control}}‐{\text{OD}}_{\text{sample}}}{{\text{OD}}_{\text{control}}}\right]\times 100. \end{array}$$

### 2.5. Determination of Inhibition of the Enzyme Beta-Hexosaminidase Method From RBL-2H3 Cells

The inhibitory effects of all extracts were evaluated using a modified approach developed by Tewtrakul et al. [20]. American Type Culture Collection (ATCC CRL-2256, VA, USA) provided the rat basophilic leukemia cell line (RBL-2H3). RBL-2H3 cells were cultured in MEM media containing 15% heat-inactivated FBS and 1% penicillin and streptomycin (10,000 units/mL of penicillin and 10,000 mg/mL of streptomycin). The cells were incubated with 5% CO_2_ at 37°C, placed at a density of 2 × 10_5_ cells/well, and incubated for 2 h. The cells were sensitized with 0.45 mg/mL of antidinitrophenyl-immunoglobulin E (anti-DNP IgE) and incubated for 24 h. Following that, the cells had a washing process using 400 *μ*L of Siraganian buffer, which is composed of 119 mM of NaCl, 5 mM of KCl, 5.6 mM of glucose, 0.4 mM of MgCl_2_, 1 mM of CaCl_2_, 25 mM of piperazine-*N*, N0-bis (2-ethanesulfonic acid) (PIPES), 0.1% of bovine serum albumin (BSA), and 40 mM of NaOH, with a pH of 7.2. Following the washing step, the cells were incubated in an additional 160 *μ*L of buffer A for 10 min. Siraganian buffer was added to dilute the stock solution. The 20 *μ*L of each concentration was placed into each well and incubated for 10 min, and then 20 *μ*L of antigen (DNP-BSA) was added to it . We transferred 50 *μ*L of the supernatant into 96-well plates and then added 50 *μ*L of a 1 mM substrate (p-nitrophenyl-N-acetyl-b-D-glucosaminide [PNAG]) in 0.1 M citrate buffer (pH 4.5). The plates were then incubated at 37°C with 5% CO_2_ for 2 h. It was stopped by adding 200 *μ*L of a stop solution that was 0.1 M of Na_2_CO_3_/NaHCO_3_ at pH 10.0. A microplate reader was used to measure the absorbance at 405 nm.

The inhibition (%) of *β*-hexosaminidase release by each sample was calculated using the following equation, and IC_50_ values were calculated from the Prism program.

(2) $$\begin{array}{c} \text{Inhibition }\left(\%\right)=\left[1-\frac{T-B1-B2}{C-B1-B2}\right]\times 100, \end{array}$$

The formula is as follows: C (control) = DNP-BSA and substrate (PNAG) without test sample, T (test) = DNP-BSA with substrate (PNAG) and test sample, B1 (Blank 1) = substrate (PNAG) only, and B2 (Blank 2) = substrate (PNAG) and test sample. 

### 2.6. Determination of Chemical Contents by the HPLC Method

The study of drug content analysis used the HPLC technique. Four compounds, pectolinarigenin from HR remedy from Sakpakdeejaroen, Juckmeta, and Itharat [21], *α*-mangostin from GM from Resomer RG 503H (acid-terminated poly [lactide-co-glycolide] [PLGA-COOH]), hydroxychavicol from *Piper betel* from Tokyo chemical industry (TCI) (Thailand), and eugenol from *Piper betel* from Sigma-Aldrich (Switzerland), were used as markers of the HMB extracts.

The HPLC column was Agilent XDB-C18, size 250 × 4.60 mm, and 5 *μ*m protected by a ZORBAX Eclipse XDB-C18 80A (4.6 × 12.5 mm, 5 *μ*m) guard cartridge. The HPLC system is equipped with a solvent pump, solvent degasser and autosampler, a G1311A quaternary solvent pump (1200 series), a G1322A solvent degasser (1200 series), a G1313A autosampler (1100 series), and a G1316A column compartment (1100 series). Photodiode array detector (DAD) was used for detection.

The elution process was conducted using gradient solvent systems at a flow rate of 1.0 mL/min under ambient temperature conditions. The mobile phase system was 0.1% orthophosphoric acid (A) in water-acetonitrile (B) using gradient elution.

All tested samples were prepared at a concentration of 10 mg/mL in methanol and filtered through a 0.45 *μ*m nylon filter. The injection volume used was 10 *μ*L of samples. Pectolinarigenin, *α*-mangostin, hydroxychavicol, and eugenol were detected at wavelengths of 284, 317, and 331 nm, respectively.

The quantitative analysis was determined using the following gradient program: At 0 min, the A: B proportion was 95: 5; at 30 min, the A: B proportion was 5: 95; at 35 min, the A: B proportion was 95: 5.

### 2.7. Statistical Analysis

The results were reported as the mean ± standard error of the mean (SEM). The IC_50_ values were calculated using the Prism software. The data were subjected to analysis using a one-way ANOVA method. The statistical significance level was set at *p* < 0.05.

## 3. Results and Discussion

### 3.1. Preparation of a Standard Calibration Curve for Quantification of HMB Extracts Using HPLC

The compound in the HMB extract was analyzed by a HPLC system. The four compounds (hydroxychavicol and eugenol found from PB Linn., pectolinarigenin found from HR remedy, and *α*-mangostin found from GM Linn.) were found at different retention times. Retention times of hydroxychavicol, eugenol, pectolinarigenin, and *α*-mangostin were 15.96, 21.46, 19.86, and 31.51, respectively.

Standard methanol solutions of hydroxychavicol, eugenol, and *α*-mangostin were prepared at concentrations ranging from 1–2000 *μ*g/mL, and standard methanol solutions of pectolinarigenin were prepared at concentrations ranging from 50–400 *μ*g/mL. Each concentration was examined three times. The standard calibration curves of hydroxychavicol, eugenol, pectolinarigenin, and *α*-mangostin were produced by plotting the peak areas versus the standard concentrations to determine their contents in the HMB extract. The linear regression equations for the determination of active compound contents are presented in the following equations:

(3) $$\begin{array}{c} \text{concentration of hydroxychavicol}=\frac{\;\left(\text{area of hydroxychavicol }–\;123.94\right)}{9.2746}\;R^{2}=0.9992,\\ \text{concentration of }\alpha \text{ mangostin}=\frac{\left(\text{area of }\alpha \text{ mangostin }–\;572.23\right)}{21.841}\;R^{2}=0.9991,\\ \text{concentration of eugenol}=\frac{\;\left(\text{area of eugenol }–\;155.93\right)}{9.2095}\;R^{2}=0.9992,\\ \text{concentration of pectolinarigenin}=\frac{\;\left(\text{area of pectolinarigenin }–\;208.56\right)}{27.282}\;R^{2}=0.9991. \end{array}$$

**Figure 1:**
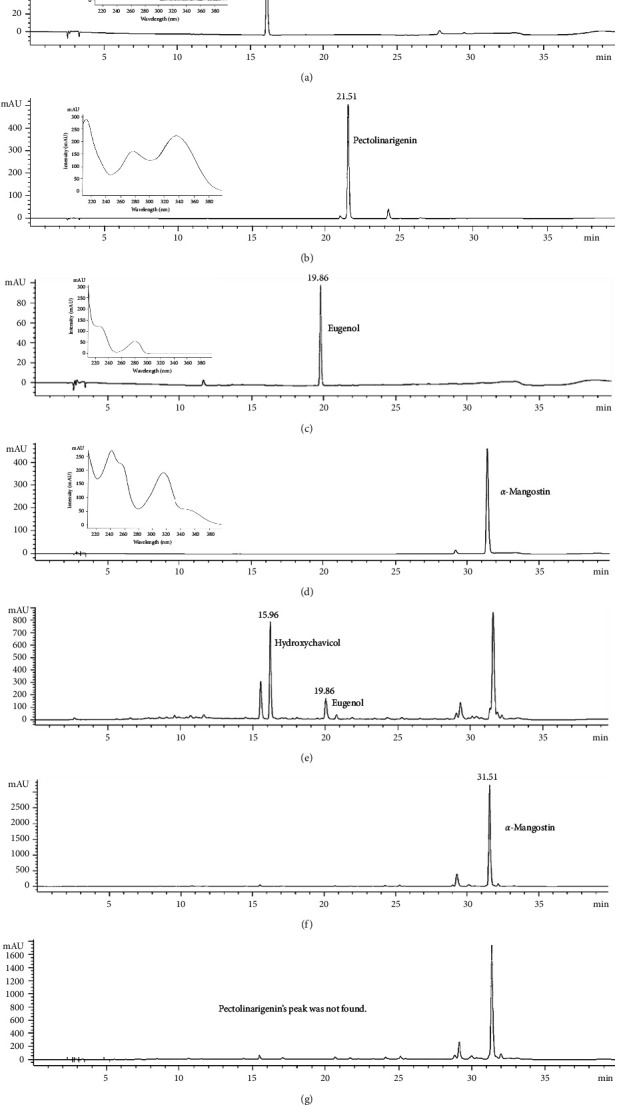
HPLC chromatogram, spectrum, and chemical structure: (a) hydroxychavicol, (b) eugenol, (c) pectolinarigenin, (d) *α*-mangostin, and (e–g) HMB at 284, 317, and 331 nm, respectively.

The resultant chromatogram of the HMB remedy extract at a wavelength of 284, 317 and 331 nm is shown in Figure 1. It was found that hydroxychavicol was the major compound in HMB extract (121.93 ± 5.74 mg/g of extract), followed by *α*-mangostin and eugenol with the content of 54.10 ± 2.36 and 86.23 ± 3.39 mg/g of extract, respectively. While pectolinarigenin was not detected in the HMB extract at both wavelengths. Therefore, hydroxychavicol, *α*-mangostin, and eugenol were used as the markers for standardizing the HMB extract.

### 3.2. Forced Degradation Study (Stress Test)

Forced degradation study, or stress test, is a technique to predict the stability of any active pharmaceutical ingredient or formulation product [22]. A drug or extract is forcefully degraded by applying artificial methods. We assessed the temperature, moisture hydrolysis, acid hydrolysis, alkaline hydrolysis, and oxidation-forced degradation tests. HPLC technique was used to identify and quantify the active compounds, and the inhibitory effects of NO production (anti-inflammatory activity) and inhibition of released *β*-hexosaminidase (antiallergic activity) that are related to AD mechanism.

**Figure 2:**
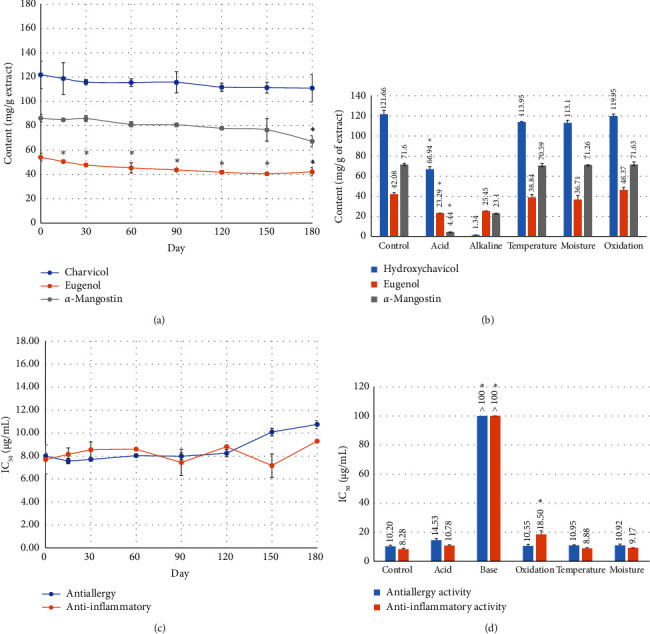
The content of three compounds in HMB remedy and inhibitory effects of HMB remedy on *β*-hexosaminidase and NO production after 6 months of storage under accelerated conditions (a, c) and after forced degradation testing (b, d). Data were expressed as the means of three determinations (*n* = 3).

The three marker contents in the HMB remedy were determined by an HPLC analytical technique. The contents of hydroxychavicol, eugenol, and *α*-mangostin in control samples were 121.66 ± 4.09, 42.08 ± 1.62, and 71.60 ± 1.11 mg/g of the extract, respectively (Table 2 and Figure 2). Temperature, moisture, and oxidation degradation samples were not significantly different in hydroxychavicol, eugenol, and *α*-mangostin contents from the control. Hydroxychavicol contents in pH degradation samples (acid and alkaline) were significantly less than control (66.94 ± 2.63 and 1.34 ± 0.09 mg/g extract, respectively). In addition, eugenol and *α*-mangostin contents in pH degradation samples (acid and alkaline) were significantly less than control (23.29 ± 3.11 vs. 25.45 ± 2.60 and 4.44 ± 0.84 vs. 23.10 ± 1.45 mg/g extract, respectively). While the contents of pectolinarigenin in all tested samples were undetectable (Table 2).

All tested samples in the forced degradation study at all tested concentrations showed > 70% cell viability. NO production inhibition in alkaline and oxidation degranulation samples had a significantly lower IC_50_ value than the control sample (> 100, 18.50 ± 2.57 and 8.28 ± 0.66 *μ*g/mL, respectively). Meanwhile, acid, temperature, and moisture degranulation samples were not significantly different from control (10.78 ± 0.64, 8.88 ± 0.37, 9.17 ± 0.14 and 8.28 ± 0.66 *μ*g/mL, respectively) (Table 3 and Figure 2). While NO production inhibition in acid, temperature, and moisture degranulation samples was not significantly different from the control.

The results of antiallergic activity by *β*-hexosaminidase assay of the HMB remedy of forced degradation study, IC_50_ values of temperature, moisture, and oxidation degranulation samples, were not significantly different from the control. While acid and alkaline degranulation samples had significantly lower IC_50_ values than control samples (14.53 ± 1.10 and > 100 *μ*g/mL, respectively). However, the acid degranulation sample still showed good antiallergic activity (Table 4 and Figure 2). While IC_50_ values in temperature, moisture, and oxidation degranulation samples were not significantly different from the control.

The HMB remedy demonstrated the susceptibility of its substance to alkaline hydrolysis. There was low anti-inflammatory activity, antiallergic activity (> 100 *μ*g/mL), and significantly decreasing contents of hydroxychavicol, eugenol, and *α*-mangostin. It was also found that contents of hydroxychavicol, eugenol, and *α*-mangostin also decreased in acid hydrolysis conditions. However, the anti-inflammatory effect was not different under acid hydrolysis conditions. The contents of the active substance were not changed under the oxidation conditions. However, it was found that the anti-inflammatory effect was slightly reduced (18.50 ± 2.57 *μ*g/mL). Both biological activities in the acid degradation sample reduced marginally with a 45% reduction in HC concentration (to 66.94 from 121.66 mg/g); however, in the alkaline condition, neither bioactivity decreased with a 98% reduction in HC content (to 1.34 from 121.66 mg/g). According to Tewtrakul, Wattanapiromsakul, and Mahabusarakam [22], the degradation of phenolic compounds including hydroxychavicol and eugenol affects many storage parameters such as light, temperature, oxygen, and pH [22]. Moreover, the *α*-mangostin has been reported to decompose in acid hydrolysis and oxidative conditions by HPLC analysis [23]. Eugenol contains functional groups that can be chemically changed, making it a suitable starting material for the synthesis of a variety of useful chemicals, one of which is hydroxychavicol [24]. It was reported that a change occurred when the PB Linn. was stored under light for 5 weeks, forming a new substance called 2,4-di-tert-butylphenol. This chemical is made when an alkylation of phenol with isobutylene takes place with acid catalysts [25]. There is a potential correlation between 2,4-di-tert-butylphenol and the eugenol degradation that occurs. The 2,4-di-tert-butylphenol production is possibly boosted by the shikimate pathway. The shikimate pathway acts as an alternative mechanism for microorganisms and plants to synthesize organic amino acids before the final reductions of coniferyl alcohol to eugenol [26, 27]. However, no peaks for other substances, particularly 2,4-di-tert-butylphenol, were seen in the tested HPLC systems.

### 3.3. Accelerated Study

The main purpose of stability testing, as outlined in the International Council for Harmonization (ICH) guideline, is to assess the effect of environmental elements, including temperature, humidity, and light, on the quality of a drug substance over a period. This evaluation is conducted to determine both the shelf life and the suggested storage conditions for the substance [18].

The accelerated testing of HMB remedy extract is to provide evidence for topical AD product development. After testing, all tested samples were tested for quantitative determination of the active substances, including hydroxychavicol, eugenol, pectolinarigenin, and *α*-mangostin by the HPLC technique and biological activity determination by NO production inhibitory assay (anti-inflammatory activity) and inhibition of released *β*-hexosaminidase (anti-inflammatory activity) that is related to AD mechanism.

1. The chemical constituents of the HMB remedy were determined by the HPLC technique. The results showed that the hydroxychavicol content in the HMB remedy showed no significant differences between Days 0, 15, 30, 60, 90, 120, 150, and 180. The contents of *α*-mangostin decreased at 180 days and *α*-mangostin content was significantly different at each period when compared to Day 0 (67.26 ± 1.65 and 71.60 ± 1.11 extract, respectively). The content of eugenol tends to decrease at 15 days and continues to decrease until 180 days. There were significant differences at each period when compared to Day 0. While the contents of pectolinarigenin in all tested samples were undetectable (Table 2).
2. The results of inhibitory activity against LPS induced NO production of the HMB remedy under the accelerated condition, and IC_50_ values of NO inhibitory effect were not significantly different between Days 0, 15, 30, 60, 90, 120, 150, and 180 (Table 3 and Figure 2).
3. The results of antiallergic activity by *β*-hexosaminidase assay of the HMB remedy under the accelerated condition IC_50_ showed no significant differences between days 0, 15, 30, 60, 90, 120, 150, and 180 (Table 4 and Figure 2)

Accelerated testing refers to a technique used to increase the rate at which chemical degradation or physical alteration of a pharmaceutical compound occurs, achieved through the application of accelerated storage conditions. There is a part of the formal stability study. The HMB remedy extract was kept under accelerated conditions of 40°C with 75% RH for 180 days, which can be inferred that the substance remains stable at standard room temperature for a duration of 2 years.

The stability study on HMB remedies demonstrated that the contents of some substances had changed. Eugenol content from Day 15^th^ to 180^th^ became lower than the starting point. Moreover, *α*-mangostin content on Day 180^th^ also became slightly lower than the starting point. Hydroxychavicol has been reported to show the best stability without degradation, whereas eugenol was moderately stable at low temperatures (5°C) and in dark conditions [28]. In the same environment, the structural difference could very well explain HC's strong stability. Almost all of the HC was retained under various temperature and light storage conditions. The hydroxyl groups in positions 1 and 2 of HC's benzene ring might have contributed to its increased stability [29]. Nevertheless, the report of Rivero and Garibay, 2019, indicated that the mangosteen peel extract containing *α*-mangostin was stable under accelerated study conditions [30]. Plants that are components of HMB also contain other substances that have anti-inflammatory activity by decreasing NO production including *γ*-mangostin, and garcinoxanthones B and C in GM showed inhibitory effects with IC_50_ values of 6.0, 11.3, and 18.0 *μ*M, respectively [31]. The efficacy could come from these substances. However, further studies may be needed. This result indicates that the HMB remedy had unaltered NO inhibitory activity when kept at accelerated conditions and could be kept under ambient storage conditions for 2 years.

It was concluded that for the stability of the extract, HMB should be stored in the refrigerator at a low temperature and kept in a closed container. HMB should be protected from various environmental conditions including light, high temperatures, oxygen, and pH to avoid the degradation of substances, especially EU.

## 4. Conclusion

The HMB remedy showed stable chemical and biological properties, with no significant differences in moisture and temperature hydrolysis. However, it was susceptible to alkaline hydrolysis and showed low anti-inflammatory activity and a significant decrease in the contents of hydroxychavicol, eugenol and *α*-mangostin. The contents of hydroxychavicol, eugenol, and *α*-mangostin decreased under acid hydrolysis conditions. The contents of active substance were not changed under the oxidation conditions. However, it was found that the anti-inflammatory effect was slightly reduced. The stability study on HMB remedies demonstrated that the contents of some substances had changed. Eugenol content from Day 15^th^ to 180^th^ became lower than the starting point. *α*-Mangostin content on Day 180^th^ also became slightly lower than the starting point. However, the reduction of the two substances did not change the anti-inflammatory and antiallergic effects. The HMB remedy's stability was maintained under ambient storage conditions for 2 years. To maintain its effectiveness, it should be stored at low temperatures and protected from various environmental conditions. Our findings provide valuable insight into the stability of this traditional remedy for future product development and its potential as a new drug for global AD treatment.

## Figures and Tables

**Table 1:** Voucher specimen number of component plants in HMB remedy and their percentage yields of crude extracts (% w/w).

Plant species/medicine	Family name	Common name	Thai name	Voucher specimen number	% Yield
*Capparis micracantha* DC.	CAPPARIDACEAE	Caper-thorn	Ching-Chi	SKP 391 03 13 01	—
*Clerodendrum petasites* S. Moore.	VERBENACEAE	—	Thao-Yai-Mom	SKP 202 03 09 01	—
*Ficus racemosa* Linn.	MORACEAE	Cluster fig tree	Ma-Duae-Chum-Porn	SKP 117 06 18 01	—
*Harrisonia perforata* Merr.	SIMAROUBACEAE	—	Khon-Tha	SKP 178 08 16 01	—
*Tiliacora triandra* Diels.	MENISPERMACEAE	—	Ya-Nang	SKP 114 20 20 01	—
Ha-Rak remedy					3.18
*Garcinia mangostana* Linn.	GUTTIFERAE	Mangosteen	Mung-Kud	SKP 214 09 13 01	21.38
*Piper betle* Linn.	PIPERACEAE	Betle	Phlu	SKP 146 16 02 01	30.16

**Table 2:** The content of compounds (mg/g) in HMB extract after forced degradation testing and 6 months of storage under accelerated conditions.

Stability study	Sample	Content (mg/g)			
		Hydroxychavicol	Pectolinarigenin	Eugenol	*α*-Mangostin
Forced degradation testing	Control	121.66 ± 4.09	ND	42.08 ± 1.62	71.60 ± 1.11
	Acid	66.94 ± 2.63⁣^∗^	ND	23.29 ± 3.11⁣^∗^	4.44 ± 0.84⁣^∗^
	Alkaline	1.34 ± 0.09⁣^∗^	ND	25.45 ± 2.60⁣^∗^	23.10 ± 1.45⁣^∗^
	Temperature	113.95 ± 0.41	ND	38.84 ± 3.11	70.59 ± 2.03
	Moisture	113.10 ± 2.73	ND	36.71 ± 4.03	71.26 ± 0.16
	Oxidation	119.95 ± 1.89	ND	46.37 ± 2.52	71.63 ± 2.66

Accelerated study	Day 0	121.93 ± 5.74	ND	54.10 ± 2.36	86.23 ± 3.39
	Day 15	118.86 ± 1.17	ND	50.60 ± 1.41⁣^∗^	84.94 ± 1.88
	Day 30	115.90 ± 1.04	ND	47.73 ± 1.06⁣^∗^	85.89 ± 0.64
	Day 60	115.55 ± 1.60	ND	45.38 ± 0.83⁣^∗^	81.18 ± 2.75
	Day 90	115.70 ± 0.94	ND	43.70 ± 0.28⁣^∗^	80.80 ± 0.61
	Day 120	111.76 ± 0.19	ND	41.80 ± 0.90⁣^∗^	77.94 ± 0.67
	Day 150	111.36 ± 1.70	ND	40.53 ± 1.01⁣^∗^	76.64 ± 1.36
	Day 180	110.92 ± 2.26	ND	42.14 ± 0.40⁣^∗^	67.26 ± 1.65⁣^∗^

*Note:* Data were expressed as the means of three determinations (*n* = 3).

Abbreviation: ND, not detected.

⁣^∗^Forced degradation testing: significant differences (*p* < 0.05) compared with control.

⁣^∗∗^Accelerated study: significant differences (*p* < 0.05) compared with day 0.

**Table 3:** Inhibition on LPS-induced NO release (%) from RAW 264.7 cells of HMB extract after forced degradation testing and 6 months of storage under accelerated conditions.

Stability study	Sample	Inhibition of NO release (%) from RAW 264.7 cells at various concentrations (*μ*g/mL)						IC_50_ ± SEM (*μ*g/mL)
		0.01	0.1	1	10	30	100	
Forced degradation testing	Control	−4.15 ± 1.42	1.14 ± 0.65	7.62 ± 1.89	74.02 ± 5.14	96.43 ± 1.93	—	8.28 ± 0.66
	Acid	−1.72 ± 3.00	3.69 ± 1.47	6.26 ± 1.44	42.45 ± 4.87	90.28 ± 0.28	—	10.78 ± 0.64
	Alkaline	—	—	—	—	−4.44 ± 1.19	15.53 ± 2.64	>100∗
	Temperature	−2.22 ± 4.10	2.26 ± 3.14	7.55 ± 2.10	66.12 ± 6.02	94.27 ± 0.93	—	8.88 ± 0.37
	Moisture	−4.25 ± 2.71	1.15 ± 1.54	2.85 ± 0.62	65.94 ± 4.16	92.09 ± 0.69	—	9.17 ± 0.14
	Oxidation	4.67 ± 1.51	6.21 ± 1.40	7.33 ± 0.99	22.04 ± 1.53	73.73 ± 2.49	—	18.50 ± 2.57∗

Accelerated study	Day 0	−3.58 ± 3.18	6.16 ± 1.11	13.59 ± 2.92	80.33 ± 3.69	96.92 ± 1.65	—	7.70 ± 1.26
	Day 15	−5.77 ± 2.40	3.89 ± 2.41	13.74 ± 4.88	84.27 ± 1.92	94.80 ± 1.82	—	8.15 ± 0.56
	Day 30	−3.82 ± 1.51	8.23 ± 1.53	24.56 ± 3.26	68.03 ± 3.73	92.83 ± 0.75	—	8.55 ± 0.69
	Day 60	−4.63 ± 0.66	5.10 ± 0.95	22.82 ± 1.75	69.08 ± 2.89	89.84 ± 0.65	—	8.61 ± 0.04
	Day 90	−5.23 ± 1.91	3.93 ± 0.41	21.86 ± 2.01	77.26 ± 5.54	95.29 ± 4.37	—	7.44 ± 1.14
	Day 120	−6.66 ± 0.86	7.40 ± 0.46	25.43 ± 2.78	76.14 ± 3.61	95.15 ± 0.85	—	8.82 ± 0.10
	Day 150	−6.36 ± 1.70	4.54 ± 1.25	24.53 ± 7.85	86.32 ± 7.14	95.20 ± 1.74	—	7.17 ± 1.02
	Day 180	−3.44 ± 1.47	11.83 ± 1.67	22.65 ± 2.12	68.86 ± 1.96	93.66 ± 0.61	—	9.30 ± 0.11

*Note:* Data were expressed as the means of three determinations (*n* = 3).

^∗^Significant differences (*p* < 0.05) compared with control.

**Table 4:** Inhibition of released *β*-hexosaminidase (%) from the antigen-induced degranulation of IgE-sensitized RBL-2H3 cells of HMB extract after forced degradation testing and 6 months of storage under accelerated conditions.

Stability study	Sample	Inhibition of *β*-hexosaminidase release (%) at various concentrations (*μ*g/mL)				IC_50_ ± SEM (*μ*g/mL)
		1	10	50	100	
Forced degradation testing	Control	4.45 ± 4.43	50.00 ± 6.52	70.54 ± 2.96	99.06 ± 1.08	10.20 ± 0.85
	Acid	0.21 ± 2.70	37.13 ± 4.01	65.35 ± 6.81	73.01 ± 3.60	14.53 ± 1.10∗
	Alkaline	—	—	24.44 ± 15.03	27.27 ± 6.60	>100∗
	Temperature	9.61 ± 16.09	46.67 ± 3.01	71.76 ± 1.09	92.08 ± 8.90	10.95 ± 0.39
	Moisture	3.74 ± 5.36	47.05 ± 5.39	78.24 ± 7.35	78.24 ± 7.35	10.92 ± 0.97
	Oxidation	9.93 ± 5.66	48.80 ± 6.34	71.50 ± 8.35	84.49 ± 3.46	10.55 ± 1.21

Accelerated study	Day 0	14.52 ± 1.50	58.75 ± 2.66	81.12 ± 2.55	89.95 ± 4.19	8.00 ± 0.26
	Day 15	9.19 ± 1.61	62.94 ± 2.91	80.11 ± 1.50	89.09 ± 2.68	7.57 ± 0.23
	Day 30	9.25 ± 1.03	61.83 ± 1.97	78.15 ± 1.42	87.03 ± 1.40	7.71 ± 0.14
	Day 60	9.47 ± 0.21	59.53 ± 0.91	78.18 ± 2.75	87.67 ± 2.09	8.04 ± 0.08
	Day 90	9.78 ± 1.37	60.00 ± 3.80	75.87 ± 1.92	89.06 ± 1.08	7.99 ± 0.30
	Day 120	10.82 ± 0.27	58.14 ± 2.90	78.57 ± 3.29	90.37 ± 1.89	8.25 ± 0.30
	Day 150	7.52 ± 2.01	49.69 ± 2.25	72.75 ± 4.74	84.91 ± 3.36	10.10 ± 0.34
	Day 180	4.45 ± 5.01	47.05 ± 2.34	74.57 ± 4.74	90.38 ± 4.26	10.76 ± 0.35

*Note:* Data were expressed as the means of three determinations (*n* = 3).

^∗^Significant differences (*p* < 0.05) compared with control (Day 0).

## Data Availability

The data used to support the findings of this study are available from the corresponding author upon request.

## Abbreviations

HR: Ha-Rak remedy
PB: *Piper betle* Linn
GM: *Garcinia mangostana* Linn
AD: Atopic dermatitis
Ig: Immunoglobulin
CAMs: Complementary and alternative medicines
NO: Nitric oxide
LPS: Lipopolysaccharide
HPLC: High-performance liquid chromatography
TTM: Thai traditional medicine
FBS: Fetal bovine serum
MEM: Minimum essential medium
P/S: Penicillin–streptomycin
DMEM: Dulbecco's Modified Eagle Medium
PBS: Phosphate buffer saline
MTT: Thiazolyl blue tetrazolium bromide
DMSO: Dimethylsulfoxide
IC_50_: Inhibition concentration at 50%
IgE: Immunoglobulin E
TNF-*α*: Tumor necrosis factor
PGE_2_: Prostaglandin E_2_
GM-CSF: Granulocyte-macrophage colony-stimulating factor
ICH: International Conference on Harmonization
